# Synapses tagged, memories kept: synaptic tagging and capture hypothesis in brain health and disease

**DOI:** 10.1098/rstb.2023.0237

**Published:** 2024-07-29

**Authors:** Mohammad Zaki Bin Ibrahim, Zijun Wang, Sreedharan Sajikumar

**Affiliations:** ^1^ Department of Physiology, Yong Loo Lin School of Medicine, National University of Singapore, Singapore 117597, Singapore; ^2^ Neurobiology Programme, Life Sciences Institute, National University of Singapore, Singapore 119077, Singapore; ^3^ Healthy Longevity Translational Research Programme, Yong Loo Lin School of Medicine, National University of Singapore, Singapore 117597, Singapore

**Keywords:** synaptic tagging and capture, synaptic plasticity, long-term potentiation, long-term depression, hippocampus, Synaptic Tagging, memory

## Abstract

The synaptic tagging and capture (STC) hypothesis lays the framework on the synapse-specific mechanism of protein synthesis-dependent long-term plasticity upon synaptic induction. Activated synapses will display a transient tag that will capture plasticity-related products (PRPs). These two events, tag setting and PRP synthesis, can be teased apart and have been studied extensively—from their electrophysiological and pharmacological properties to the molecular events involved. Consequently, the hypothesis also permits interactions of synaptic populations that encode different memories within the same neuronal population—hence, it gives rise to the associativity of plasticity. In this review, the recent advances and progress since the experimental debut of the STC hypothesis will be shared. This includes the role of neuromodulation in PRP synthesis and tag integrity, behavioural correlates of the hypothesis and modelling *in silico*. STC, as a more sensitive assay for synaptic health, can also assess neuronal aberrations. We will also expound how synaptic plasticity and associativity are altered in ageing-related decline and pathological conditions such as juvenile stress, cancer, sleep deprivation and Alzheimer’s disease.

This article is part of a discussion meeting issue ‘Long-term potentiation: 50 years on’.

## 1. Introduction

Every day, we encounter various experiences, each inundated with information. Yet most information and experiences are lost in the sands of time, while others persist as enduring memories. Understanding the neurobiological underpinnings of how memory is encoded, retained and lost remains a central question in research exploring neuronal functions in health and disease.

It is understood that memories are stored in representations known as engrams or traces across different brain regions. These representations require the persistent change of connectivity between neurons and regions. Connectivity between neurons, and in turn brain regions, is mediated by synapses, which are biochemically distinct structures. Chemical synapses employ neurotransmitters to relay information between neurons, and this is referred to as synaptic efficacy. The ability of synapses to change their efficacy is known as synaptic plasticity [1].

**Figure 1.**
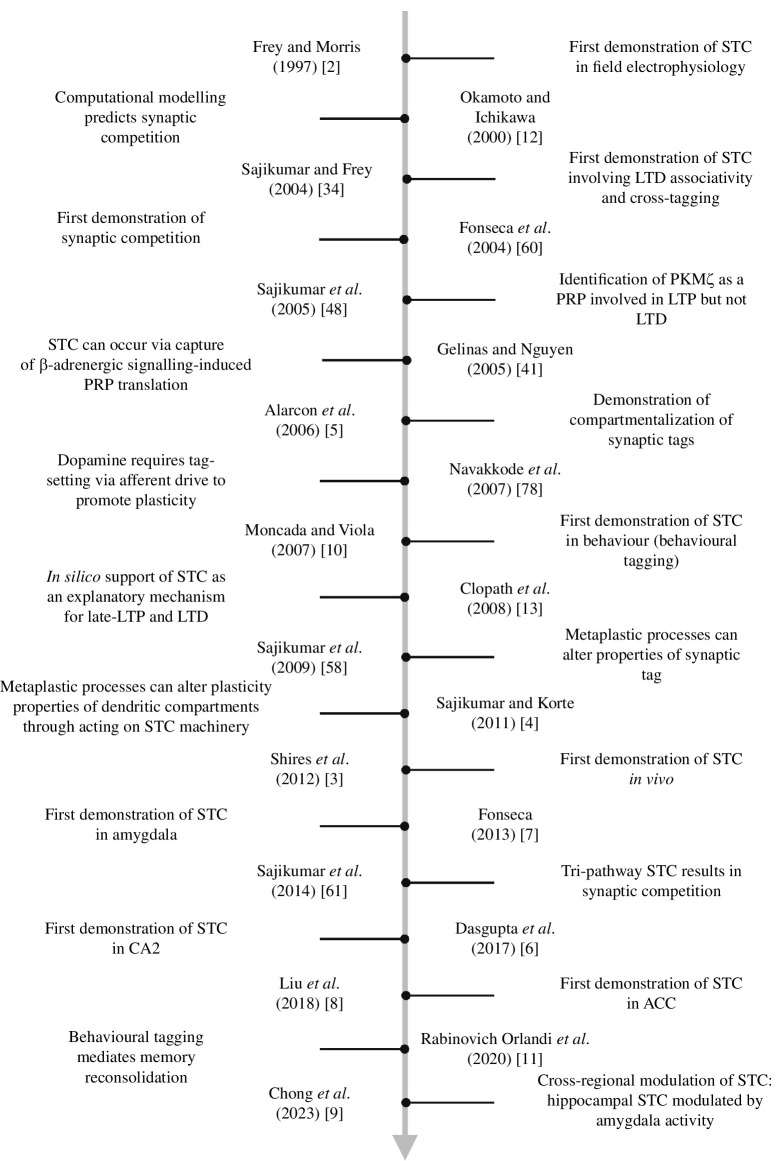
Timeline depicting key studies in understanding fundamental properties of STC [2,3] and how STC has been used in different ways to inform on synaptic plasticity processes. These include investigations on the plasticity-related product (PRP) synthesis, compartmentalization [4,5] and regions beyond the CA1 [6–9]. Key behavioural [10,11] and computational [12,13] findings are also highlighted. ACC, anterior cingulate cortex; LTD, long-term depression; LTP, long-term potentiation.

To effect persistent synaptic modification, several cellular mechanisms need to occur. The synaptic tagging and capture (STC) hypothesis offers a conceptual framework on cellular consolidation of synaptic plasticity. Moreover, STC also permits memory associativity via heterosynaptic interactions. In this review, we will share how our understanding of STC has evolved over the years, and in turn enhanced our knowledge of memory processes. A timeline of key studies in understanding STC is detailed in figure 1. In the second half of the article, we will discuss how it serves as a model to elucidating synaptic deficits in ageing and other pathological conditions.

## 2. Synaptic tagging and capture: governing persistence of long-term potentiation

As a model of synaptic plasticity, long-term potentiation (LTP) occurs when synapses exhibit a prolonged heightened response after a high-frequency afferent stimulation [14]. LTP is regarded as the cellular correlate of memory in the brain [1,15]. LTP may be expressed in a transient or persistent form, early-LTP and late-LTP, respectively [16,17]. In early-LTP, synapses exhibit potentiation but return to their pre-stimulation levels within minutes to hours. When the potentiation remains persistent over hours and days, and even months, the synapses have achieved late-LTP [18–21]. Other reviews share more details on different phases of LTP [17,22,23].

What determines the persistence of late-LTP is the activation of protein synthesis [20,24]. Protein synthesis following synaptic activity will generate plasticity-related products (PRPs), a catch-all term for proteins and molecular players that promote long-lasting LTP [2].

A significant proportion of neuronal PRP synthesis processes, such as mRNA synthesis and protein translation, takes place within the soma [25]. Yet, LTP strengthening occurs in synapses. The distance between soma and synapse may span hundreds of microns, a non-trivial distance for subcellular transport. Furthermore, neurons host many synaptic junctions, which in turn are connected to different afferents. The incorrect ‘delivery’ of the PRPs is detrimental to cell energetics and can lead to errors in neuronal computation. How does the neuron ensure that these products are transported to the intended synapse?

Thus, Frey and Morris [2] proposed the following: when a synapse receives sufficient activity, it will activate signalling cascades that propagate to the soma, activating PRP synthesis. The activated synapses will also present a transient tag to capture the newly synthesized PRPs. This forms the main premise of the STC hypothesis [26].

To verify this hypothesis, they conducted two-pathway field electrophysiology experiments on acute rat hippocampal slices, where the synaptic efficacy of two independent Schaffer collateral synaptic inputs converging onto the same neuronal population in the subfield CA1 was monitored [27,28]. The first pathway was activated via strong tetanization, leading to protein synthesis-dependent LTP. After 1 h, the second pathway was then strongly tetanized in the presence of protein synthesis blocker anisomycin. Typically, perturbing *de novo* protein synthesis disrupts PRP generation and hence late-LTP expression. However, both pathways exhibited late-LTP. Apparently, synapses in the second tetanized pathway still presented tags that captured PRPs whose synthesis was induced by the first tetanized pathway. Thus, STC expanded plasticity studies from a homosynaptic to a heterosynaptic perspective.

**Figure 2.**
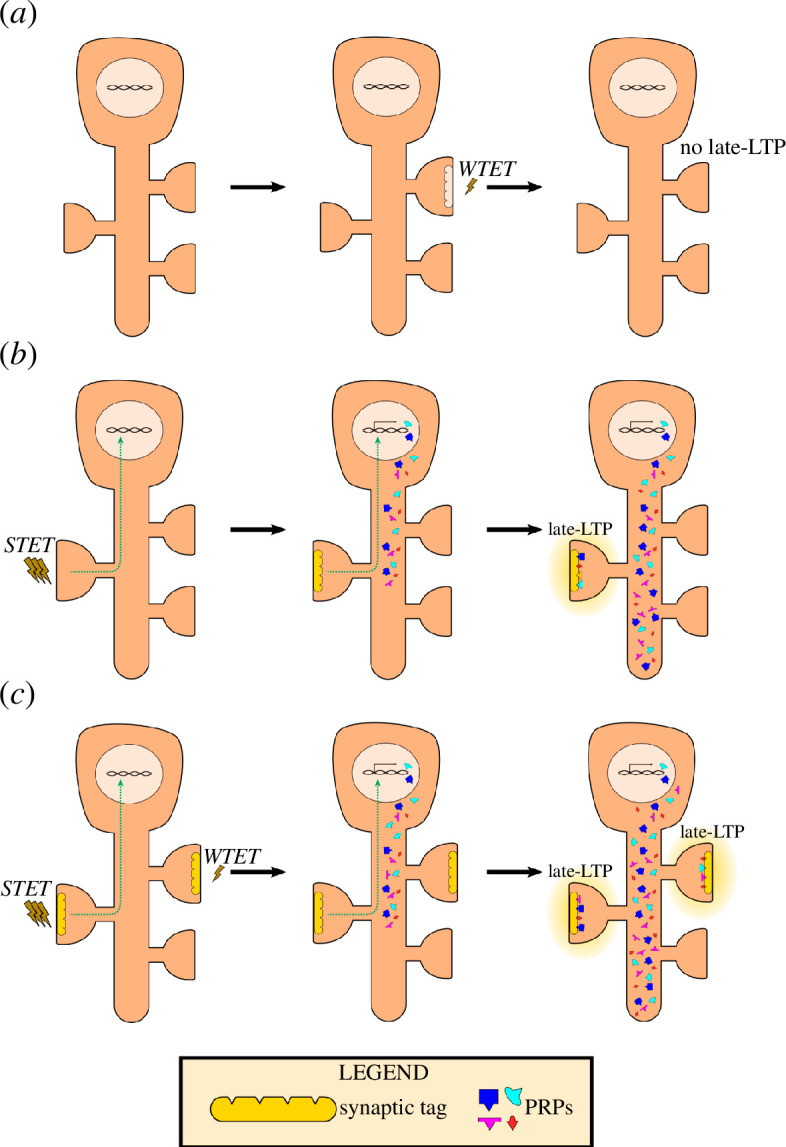
Persistence and associativity of plasticity require STC mechanisms. (*a*) Weak stimulus, that is weak tetanization (WTET), induces tag setting but not activation of signalling pathways towards PRP synthesis. Thus, the tetanized synapse loses its transient tag and does not exhibit long-lasting potentiation. (*b*) Strong stimulus (STET) is delivered onto a synapse. This will trigger tag setting and PRP synthesis through signalling cascades (green non-continuous line). PRPs are made available in the neuron and are captured by the tag present in the tetanized synapse. As such, the synapse maintains an input-specific long-lasting LTP. (*c*) Weak and strong stimuli in temporal proximity (WTET of a synapse occurs in temporal proximity of STET of another synapse). STET induction leads to PRP synthesis. These PRPs are then captured by both tags set by the WTET- and STET-activated synapses. Thus, both synapses exhibit long-lasting potentiation. This is a graphical representation of how associativity of synapses is achieved through STC.

Interestingly, through STC, weakly activated synapses that alone express early-LTP can achieve persistence in the presence of PRP availability triggered by strongly activated synapses. As long as PRPs are made available during the tag presentation, which lasts roughly 2 h, weakly activated synapses can demonstrate late-LTP [29]. Experimentally, this has been shown both ways: using the strong-before-weak protocol, where a strong stimulation is given to one synaptic pathway first, then a weak stimulation given to another pathway, or using the weak-before-strong protocol, where the two manipulations are temporally swapped [2,29]. In short, the associative property of synaptic interactions is a direct consequence of STC (graphical representation in figure 2). Hence, STC has been the prevailing model for examining interactions between populations of synapses and, in turn, how different forms of memories associate with one another and achieve persistence.

### (a) STC for long-term depression

Long-term depression (LTD), the activity-dependent diminution of synaptic strength, is another model of plasticity [30–32]. Its expression is crucial for reversal learning and behavioural flexibility in response to environmental changes.

LTD is expressed homosynaptically, indicating input specificity [31,32]. Furthermore, it requires protein synthesis [33]. Thus, STC could also govern the expression of LTD. Replicating the seminal synaptic tagging study, Sajikumar and Frey [34] revealed that LTD-induced synapses maintain their long-lasting plasticity through the STC mechanism. Thus, STC has emerged as the mechanism underlying long-lasting plasticity of both polarities: depression and potentiation.

### (b) Induction protocols, tag and PRPs in LTP and LTD

The induction protocol dictates the polarity of the plasticity event in the synapses. This polarity is manifested through molecular and structural changes in the synaptic environment and the tag setting [22]. Changes within synapses subjected to plasticity induction contribute towards the tag setting process. The tag set, which can be either LTP- or LTD-specific, will recruit PRPs that promote the persistence of the plasticity event. PRPs involved in the synaptic modification can also be process-specific.

For LTP induction, a high-frequency protocol of 100 Hz is often used. This induces rapid calcium transients into the intracellular space, activating calcium-dependent kinases such as protein kinase A (PKA) and calcium/calmodulin-dependent protein kinase II (CaMKII), promoting LTP [35–38]. Signalling pathways recruited for LTP also include cyclic adenosine monophosphate (cAMP)-associated pathways, cAMP response element-binding protein (CREB) and extracellular signal-regulated kinases 1/2 (ERK1/2) [39–41]. This will lead to the expression of PRPs, such as zeta isoform of atypical protein kinase C (PKMζ) and brain-derived neurotrophic factor (BDNF) [42,43]. Upon expression, these PRPs stabilize more α-amino-3-hydroxy-5-methyl-4-isoxazolepropionic acid receptors (AMPARs) at the postsynaptic membrane and improve their conductance via phosphorylation to sustain LTP expression.

LTD is instead induced via low-frequency stimulation (1 Hz) onto synapses, which results in slower but more sustained calcium transients. This calcium influx leads to the activation of calcium-dependent phosphatases and the dephosphorylation of LTP-associated pathways. Signalling pathways involving protein kinase C (PKC), p75 neurotrophic factor (p75^NTR^) and postsynaptic serine/threonine protein phosphatase 1 (PP1) have been implicated in LTD. These processes promote the synthesis of LTD-specific PRPs such as the immature form of BDNF (proBDNF), activity-regulated cytoskeleton-associated protein (Arc) and signalling proteins such as calcineurin and other calcium-dependent phosphatases [44–46]. By facilitating the trafficking of AMPARs away from the postsynaptic density, they maintain LTD expression [46].

### (c) Cross-tagging: positive associative interactions of LTP and LTD

The different forms of plasticity, LTP and LTD, as mentioned earlier, are associated with different putative proteins. Thus, these plasticity players can be considered process-specific [47]. However, synaptic activity triggers the synthesis of a pool of various PRPs that support both forms of plasticity [34]. This is demonstrated in cross-tagging, where PRPs synthesized in response to one form of plasticity (LTP/LTD) can be captured by synapses activated for a different form of plasticity (LTD/LTP).

Further investigations reveal the identity of these process-specific PRPs. First, there is PKMζ, which is necessary for LTP maintenance but not for LTD [43,48]. For LTD-specific PRPs, calcineurin and Arc were identified [46,49]. A few have shown process independence, such as BDNF. Moreover, it is noteworthy that even though different induction protocols recruit different enzymatic players in its signalling cascade, they generally lead to the generation of a common pool of PRPs that can serve both processes of synaptic modification, as seen in cross-tagging experiments. This suggests a convergence of signalling pathways.

However, the pool of PRPs synthesized can be biased to promote only one form of plasticity, and this can be determined by the type of induction. Most studies discussed thus far involve pre-synaptic stimulations of projecting axons. However, another protocol that pairs presynaptic and postsynaptic responses, known as spike-timing-dependent plasticity (STDP), can promote synaptic associativity via STC, but cannot facilitate cross-tagging across synapses [50]. Thus, there may be mechanistic differences in the signalling pathways induced by STDP that differ from other induction protocols. Understanding this difference will also shed light on how different kinds of synaptic activity can influence the process specificity of synthesized PRPs and consequent neuronal computation.

### (d) Elucidating and characterizing the synaptic tag

While the tag serves as a conceptual placeholder, efforts have been made to pinpoint the structural profile of the tag. Tag candidates must meet certain criteria, as outlined by Redondo & Morris [51]. First, its activation must be confined in a synapse-specific manner. Its active state must also be transient and reversible. Finally, it must interact with other PRPs to promote plasticity. Through these criteria, several candidates for the tag have been suggested, including calcium-permeable AMPARs, CaMKII, tropomyosin-related kinase B (TrkB), actin architecture and PKA [52–55]. Some of these candidates have dual roles as PRPs and signalling molecules that promote PRP synthesis. However, owing to the challenge of pharmacologically targeting these molecules without affecting cellular energetics or architecture, it remains difficult to prove their role as tag molecules conclusively.

Nonetheless, it appears that the tag is less likely to involve only a singular protein; instead, it could refer to the state of a synapse [51]. It is likely a protein complex that recruits several PRPs and signalling cascades to induce and promote the observed electrophysiological changes [51,56]. This complex includes scaffolding proteins, which may also involve transmembrane proteins that maintain the temporary state of active tags, ready to capture PRPs. Upon sufficient PRP interaction, it retains a stable state that sustains the plasticity change.

The nature of the tag also undergoes changes after induction and interaction with PRPs. This aspect is explored during depotentiation studies, where the tetanized synapses are immediately depotentiated by low-frequency stimulation, effectively ‘resetting’ the tags [57]. The succeeding depotentiation leads to a loss of LTP expression. However, this depotentiation needs to occur within a time window of 5 min. Low-frequency stimulation initiated 10 and 15 min after LTP induction will not prevent LTP expression. Thus, it is suggested that the tag is at a more ‘fragile’ state in its initial moments. The initial phase will involve calcium-dependent kinases activated owing to the influx of calcium, such as CaMKII. A more ‘stable’ form of the tag may involve readily available PRPs and this may also depend on the kinetics of ‘assembling’ the protein complex that constitutes the tag. The stability and longevity of this tag can also be modulated by other molecular players, indicating their influence in exerting a metaplastic effect. One such molecule is the ryanodine receptor (RYR). When RYRs are activated, synapses that undergo short-term potentiation (STP) are tagged, even though STP is typically too weak to set tags [58]. Therefore, RYR-primed STP-induced synapses can achieve long-lasting potentiation when they capture PRPs from other strongly tetanized synapses. RYR priming also renders depotentiation on tetanized synapses ineffective after a transient depotentiation [59]. It has been demonstrated that RYR priming allows tags in synapses to recruit PKMζ. Furthermore, RYR priming also extends the lifetime of tags to approximately 4 h.

While the exact structure of the tag continues to elude us, examining the nature of tag will be essential, as promoting its lifespan may be crucial in disease-associated impairments and enhancing learning. On the other hand, its transient nature may also serve a computational role in generalizing and discriminating different streams of memories.

### (e) Synaptic competition: flipside of synaptic cooperation in STC

PRP synthesis levels can also be manipulated. Limited protein synthesis, for instance, owing to the presence of anisomycin, will lead to competition between synapses [12,60]. Additionally, when three synaptic populations are simultaneously activated, they will compete for the same pool of PRPs [61]. This will also lead to synaptic competition, which may not allow for any of the tetanized populations to achieve late-LTP. A ‘winner-take-all’ phenomenon occurs, where earlier-potentiated synapses capture available PRPs, leaving later-potentiated synapses with an insufficient supply [12,61].

### (f) STC extending beyond the Schaffer collateral inputs into CA1

Although STC has been extensively studied in hippocampal CA1 neurons, it could also be involved in other brain regions and neuronal types. Indeed, STC has been shown to be the mechanism behind LTP in other hippocampal subregions and brain regions, each with unique roles beyond declarative memory. As of late, STC has been investigated in other regions such as the amygdala, hippocampal CA2 and anterior cingulate cortex [6–8,62–64]. This suggests that STC has a more universal role in synaptic learning.

First, STC has been characterized in the lateral nuclei of the amygdala [7,64]. Here, neurons receive innervations from thalamic and cortical inputs, which have different receptors—pre- and postsynaptic—that may govern tag setting [65]. These inputs are implicated in sensory and cognitive processes, respectively. Associativity of these two inputs drives classical conditioning and fear processing in the amygdala [66,67]. Hence, this body of work may suggest that STC is involved in associating cues and stimuli, as seen in reinforcement learning.

Hippocampal area CA2 has also been studied for plasticity interactions [68,69]. CA2 neurons receive two major inputs from different origins: inputs from CA3 via Schaffer collaterals and entorhinal cortical inputs [68,70]. These two inputs display different plasticity rules. For instance, Schaffer collateral inputs to CA2 are plasticity resistant, while entorhinal cortical inputs can exhibit plasticity [68,70]. These inputs to CA2 have different functions: entorhinal cortical inputs are involved in social recognition whilst the Schaffer collateral inputs are suggested to be involved in regulating overall hippocampal dynamics [71–73]. Notably, Schaffer collateral inputs to CA2 are amenable to various neuromodulators, suggesting that hippocampal computations can be modulated by neuromodulators through CA2 [74]. As these two inputs are associated with different forms of memories—episodic for Schaffer collaterals and social for entorhinal cortical inputs—CA2 could be involved via STC to permit synaptic interactions between both inputs, and in turn, memory interactions between episodic and social memories.

STC has been recently characterized in the anterior cingulate cortex (ACC) [8]. STC mechanisms within ACC were impaired in mice following tail amputation. The ACC is involved in pain perception, as well as in executive functions such as decision-making [75,76]. While further investigation is still needed, understanding the role of STC in ACC could provide insights into how peripheral injury, in the form of the tail amputation, could confer computational deficits to cognitive processes in the cortex.

Elucidating STC mechanisms in other brain regions is the nascent ground for exciting research. First, these other regions may have converging inputs from different origins and can become interesting models for studying the associativity of synapses from different inputs and how their STC interactions differ from those between Schaffer collateral inputs in CA1. Additionally, neurons from these regions receive inputs that have different functional roles. With the emerging role of STC beyond CA1, it could play a role in integrating different forms of memories through synaptic interactions. Overall, studying STC in different regions will further provide insight on how computational mechanisms of distinct neuronal populations manifest physiologically and contribute to the development of associative properties in learning and behaviour.

### (g) Neuromodulation and STC

Other neuromodulators have also been implicated in the maintenance of late-phase plasticity and their role can be explained via STC. One such neuromodulator is dopamine, a monoamine transmitter involved in reward-motivated behaviour [77]. The hippocampus also receives major dopaminergic innervation from the ventral tegmental area (VTA) and locus coeruleus (LC). Pharmacological blockage via specific D1 antagonist SCH 23390 during LTP induction prevented long-term plasticity, and the application of D1/D5 receptor agonist SKF38393 caused slow-onset potentiation [78]. Interestingly, this slow-onset potentiation of synaptic responses requires an afferent synaptic drive, highlighting the synergistic role that D1/D5 receptors play in promoting plasticity with the glutamatergic *N*-methyl-d-aspartate (NMDA) receptors. The D1/D5 receptor activates the ERK1/2 pathway, which leads to upregulation of PRPs. Thus, in the presence of SKF38393, synaptic cooperation occurs instead of synaptic competition. As dopamine is released during saliency, this molecular phenomenon explains why weak forms of memories can persist in the presence of novelty [79].

Other modulatory events can promote increased availability of PRPs, such as Group I metabotropic glutamate receptor (mGluR) activation [4]. Group I mGluR activation recruits other signalling molecules, such as the mechanistic target of rapamycin (mTOR), to promote increased PRP synthesis.

Additionally, other neuromodulators associated with affective moods can also promote plasticity through STC. For instance, typically aplastic Schaffer collateral inputs within the CA2 are susceptible to modulation by substance P, a peptide neuromodulator associated with pain. In the presence of substance P, these inputs exhibit protein synthesis-dependent slow-onset potentiation that permits associativity of weakly activated entorhinal cortical inputs [6]. CA1 plasticity can also be modulated by the basolateral amygdala (BLA), which is involved in emotional and valence processing [9]. Interestingly, BLA activation can promote plasticity in CA1 by increasing PRP synthesis, which also promotes synaptic cooperation. However, excessive BLA activation impedes plasticity altogether in CA1 by reversing and reducing PRP availability. The work involving mood-associated neurotransmitter and BLA activation underlines the role of emotional cues in regulating the plasticity of episodic memories through STC mechanisms of hippocampal neurons in both health and disease.

### (h) Behavioural tagging: behavioural correlate of STC

Beyond plasticity studies, STC has been extended into the realm of behaviour through a process known as behavioural tagging. Briefly, behavioural tagging occurs when a transient memory achieves memory persistence owing to the proximity of its encoding to a strong memory encoding event [10,80]. In the foundational study, rats were exposed to a novel open field, which leads to PRP synthesis in hippocampal neurons. Subsequently, a transient hippocampal-dependent memory, such as distinguishing a subtle change in object location or recalling a weak footshock event, was encoded in close temporal proximity, resulting in persistence of the said memory. This occurs because the tags for the transient memory capture PRPs synthesized by the strong novel open-field memory. The team showed that temporal proximity is necessary, and that both strong and weak memories must be encoded within the same brain region to interact.

Behavioural tagging can be observed outside the laboratory through the phenomenon of ‘flashbulb’ memories, in which seemingly trivial memories that are temporally adjacent to traumatic or impactful events are remembered vividly. For instance, one may forget the details of last Tuesday’s lunch, but can vividly recall their meal during their graduation, marriage or other significant life event. Such ‘flashbulb’ memories are also observed in survivors of major tragedies. STC provides a neurobiological framework for understanding this phenomenon, where salient memories cause the persistence of otherwise trivial memories that occur within their associative timeframe.

Behavioural tagging also has similar characteristics to that of STC: it is protein synthesis-dependent and involves tag transience in weak memory [80]. The co-release of modulatory neurotransmitters such as dopamine and noradrenaline also plays a role in behavioural tagging, similar to STC [81]. While dopamine co-activation is shown to be necessary for memory associativity, it can also promote memory persistence in the CA1 through the LC [82,83].

Furthermore, most *ex vivo* electrophysiological studies primarily focus on events occurring during the initial stages of memory encoding. Yet, there is still much to learn about memory processing beyond the initial encoding and consolidation phases, where memory traces are not necessarily permanent and can become labile upon reactivation [84]. Behavioural tagging experiments demonstrate that reactivated memory traces, which can occur owing to re-exposure to the trained environment or conditioned stimulus, subsequently undergo protein synthesis-dependent re-consolidation, involving PRPs such as PKMζ [85]. This requirement for protein synthesis suggests that re-consolidation could also involve STC mechanisms [11]. Further behavioural tagging experiments can be conducted during reactivation and reconsolidation to improve our understanding of the molecular events behind these processes.

### (i) Computation work in STC

The STC framework also presents exciting ground for computational *in silico* work. Physiological work informs computational studies, which provide valuable insight into how synapses persist over time and offer predictive models of synaptic rules and behaviour.

These models, in turn, can be validated by physiological studies. Such work has played a pivotal role in shaping key concepts in STC, including synaptic competition [12], the constant synthesis of PKMζ to sustain memory traces [86] and the notion of a trigger or threshold phenomenon that must be achieved for activated synapses to achieve long-lasting plasticity from transient forms [13]. This latter model received further support through the discovery of other modulatory molecules that reduce the threshold to promote plasticity [58,87,88].

Recent computational work has confirmed the significance of neuromodulatory events in memory consolidation via STC [89]. Computational models can also shed light on how STC is involved in consolidating overlapping memory representations [90], bridging the conceptual gap with other learning processes such as schema consolidation [91]. Both physiological and computational models play reciprocal roles in advancing our understanding of STC, with computational models being instrumental in predicting the next questions that physiological experiments can aim to answer [92].

## 3. STC in pathologies

In the second half of this review, we will discuss several physiological disorders and their unhealthy impact on hippocampal synaptic plasticity. We will pay particular attention to the potential novel insights that can be gained by interpreting established results using the STC model or by conducting experiments that directly investigate the STC phenomenon in pathological states.

### (a) Juvenile stress

It is well established that stress during development has chronic effects on cognitive functions related to learning and memory [93]. In this context, we will specifically focus on the impact of stress during the juvenile stage, which occurs in the period immediately after weaning (~P20–P30), on learning and memory in adulthood. Research has shown that mice subjected to juvenile stress demonstrate impaired hippocampus-dependent spatial and avoidance learning in adulthood [94–96]. Interestingly, synaptic plasticity studies show variable effects of juvenile stress on hippocampal synaptic plasticity. It has been reported that juvenile stress results in reduced basal synaptic efficacy, a decreased magnitude of LTP in the dorsal CA1 region and an enhanced LTP effect in ventral CA1 in adult animals [97,98]. However, another study suggests that the effects of juvenile stress on LTP and LTD do not last, but instead facilitate persistence of the effects of subsequent adult stress on synaptic plasticity [99]. Most studies implicate dysregulation in neuromodulatory, astrocytic or inhibitory circuitry as the mechanical cause of these plasticity impairments [100,101]. While these effects are indeed strongly associated with both stress and hippocampal synaptic plasticity, it would be pertinent to understand the specific consequences of these varied factors on the molecular mechanisms of plasticity within hippocampal pyramidal neurons.

It has been shown that juvenile stress has no observable effects on the expression of LTP in CA1 [102]. However, using a weak-before-strong STC protocol, these researchers demonstrated that temporally proximal strong stimulation at one synaptic input does not allow for the expression of late-LTP at a different, nearby weakly stimulated synaptic input in juvenile stressed rats. Behavioural data, using a behavioural tagging paradigm, support these findings by indicating that juvenile stressed rats do not exhibit enhanced memory after novelty exposure. On a molecular level, these results are associated with a reduction in CREB-binding protein (CBP, an acetyltransferase), BDNF and histone deacetylase 3 (HDAC3) expression, along with enhanced expression of the G9a/G9a-like protein (GLP) epigenetic complex (primarily a histone methyltransferase). An earlier study has shown that G9a/GLP inhibition has an LTP-priming effect, and this LTP is able to participate in STC [103]. G9a/GLP enhancement can also lead to decreased expression of PKMζ [104]. Collectively, these findings propose a molecular pathway that is responsible for some of the observed learning and memory deficits in juvenile stressed mice, which may be downstream of glucocorticoid-dependent mechanisms of stress. While future studies will be needed to confirm the connection between known neuromodulatory or glial mechanisms and these epigenetic findings, this analysis of associative plasticity has provided a potential piece of the puzzle in understanding juvenile stress-induced synaptic plasticity dysregulation.

### (b) Sleep deprivation

Sleep has been associated with the consolidation of new episodic and procedural memories, although the exact mechanism by which such consolidation occurs is still unconfirmed [105,106]. Conversely, when sleep is disrupted or experimentally deprived, memory processes are disturbed such that novel learning is impaired [107,108]. It is well established that sleep deprivation negatively impacts hippocampal synaptic plasticity in terms of diminished LTP induction and loss of LTP maintenance [109–113]. This has been associated with decreased glutamate receptor subunit GluR1 levels and ERK activity in the dorsal hippocampus, impaired cAMP signalling, and also with the disruption of mTOR-mediated protein synthesis, among other mechanisms [113–115].

Ted Abel’s group was the first to demonstrate the effects of sleep deprivation on the STC process [116]. In order to overcome the lack of LTP in sleep-deprived animals, they employed a massed four tetanus train LTP induction protocol, a very strong stimulus previously shown to induce LTP even in sleep-deprived animals [113]. Subsequent weak stimulation in a nearby synaptic input only resulted in the expression of early-LTP in sleep-deprived mice. The natural next step here would be to investigate how the STC mechanism has been perturbed. If we consider the earlier proposed mechanisms, GluR1, ERK and cAMP/PKA signalling have been associated with synaptic tag setting processes, while mTOR is crucial for synthesizing proteins that play the role of PRPs and are involved in LTP maintenance [47,54,117]. Further research has then demonstrated that these effects of sleep deprivation on STC correlate with the animals’ behaviour and are p75^NTR^-dependent [118]. Interestingly, p75^NTR^ has been implicated upstream of many of the identified plasticity mechanisms, exerting an influence on cAMP/PKA/LIM domain kinase 1 (LIMK1), cAMP/CREB (common downstream with ERK/mitogen-activated protein kinase (MAPK) pathway) and mTOR-related mechanisms [118,119]. Furthermore, sleep deprivation has been associated with epigenetic alterations, while STC is known to be modulated by epigenetic changes [103,120,121]. Using suberoylanilide hydroxamic acid (SAHA), an HDAC inhibitor, Wong *et al*. [122] were able to demonstrate that the effects of sleep deprivation on ERK are mediated by epigenetic modifications via histone deacetylation. Importantly, both interventions (p75^NTR^ genetic knockout and SAHA) were able to rescue STC deficits in the sleep-deprived hippocampus.

While the STC model has mainly been used to explain memory deficits resulting from sleep deprivation, some researchers hypothesize that it may also have explanatory power for the role that sleep itself plays in memory consolidation [123]. However, experimentation will be necessary to validate these ideas.

### (c) Cancer-related cognitive impairment

Advances in cancer research have significantly improved the prognosis of cancer patients in recent years. As a result, there have been increased reports of cancer-related cognitive impairment (CRCI), which describes cognitive impairments, including memory deficits, in non-central nervous system (CNS) cancer patients [124]. While it is suggested that cancer treatment strongly influences the occurrence of these impairments, pathology itself still affects cognition, albeit to a smaller degree [124,125]. There are reports of multiple forms of non-CNS cancer being associated with cognitive impairment before treatment, with animal studies showing that induction of a non-CNS tumour is sufficient to cause hippocampal-dependent memory impairments [126–130]. Understanding the cancer-related effects on hippocampal synaptic plasticity before cancer treatment and the underlying molecular mechanisms behind these clinical observations will be essential in managing CRCI. Unfortunately, there is a lack of such studies within the literature.

Recently, results of synaptic plasticity investigations in liver carcinoma using a murine hepatocellular carcinoma tumour model were published [131]. As LTP in tumour-bearing mice was not disrupted, the researchers then studied the expression of associative plasticity through STC, which is more sensitive to molecular dysregulation as compared to LTP. Interestingly, using the strong-before-weak protocol, weakly stimulated synaptic inputs in tumour-bearing mice were unable to express late-LTP, even in close temporal proximity to strong stimulation at another nearby synaptic input [130]. Zhu *et al*. [131] then discovered that gut microbiota disruptions are necessary to cause these plasticity effects through STC experiments in antibiotic-treated mice and established its sufficiency using healthy mice with transplanted gut microbiota from tumour-bearing mice. This study demonstrates the potential of complementing synaptic plasticity studies with associative plasticity experiments for greater sensitivity in detecting plasticity deficits associated with memory impairments.

### (d) Ageing

Age-related cognitive impairments have been extensively studied. Notably, it is well-established that aged animals have worse learning and memory capabilities as compared to young ones [132–135]. In terms of synaptic plasticity, it is noted that LTP induced in aged animals has a diminished magnitude in the late phase compared to young animals, and that this reduction is correlated with memory impairments in the aged animal [135]. Furthermore, aged hippocampal slices exhibit increased sensitivity to LTD-inducing stimulation, such that even weak low-frequency stimulation can cause late-LTD [136,137]. Aged hippocampal neurons also appear to employ different mechanisms for initiating LTP, with a reduced contribution by NMDA receptors to postsynaptic calcium and altered calcium dynamics [138,139]. Neuromodulatory pathways that influence synaptic plasticity are also altered in ageing [140]. In particular, the dopaminergic pathway is diminished owing to reduced expression of dopaminergic receptors in the hippocampus [141]. Further details on many other changes in the plasticity properties of the aged synapse can be found in in-depth reviews on this topic [142–144].

Investigations into associative plasticity within the aged hippocampus reveal that STC does not occur in aged brains. In CA1, applying a strong-before-weak protocol in aged animals does not enable the expression of late-LTP in weakly stimulated synapses even with temporally proximal strong stimulation at other nearby synaptic pathways [145]. Similar results were observed in aged mice during weak-before-strong experiments, as well as in the anterior cingulate cortex of middle-aged animals [146,147]. Attempts to induce STC using strong dopaminergic stimulation, in which three pulses of a dopamine receptor agonist are used to induce late-LTP in a lightly stimulated (test stimulus only) pathway followed by a weak tetanus train at an initially silenced pathway, did not lead to late-LTP induction at both synapses [145]. Behaviourally, novelty-induced behavioural tagging was not observed in older animals [137,148]. Different interventions targeting molecular signalling imbalances caused by ageing during STC experiments were able to rescue associative plasticity lost in ageing. These interventions include chelation of excessive zinc, p75^NTR^ knockout, HDAC3 inhibition and activation of BDNF/TrkB signalling [137,145–147].

Considering that the threshold between LTP and LTD is shifted towards LTD, and that STC is not expressed, it is likely that impairments exist in the synaptic tagging event. This can result in failure for a late-LTP-compatible tag to be established with weaker stimulations. Some evidence supports this: since NMDA receptor signalling is impaired in ageing, immediate downstream molecular players implicated in tag setting (e.g. CaMKII, PKA) would also have reduced responses to synaptic events [149]. Zinc is known to modulate NMDA receptors negatively, and its excess in aged neurons could possibly contribute to poor NMDA receptor signalling, impairing tag setting. On the other hand, the other reported experimental interventions point towards the capability for enhanced PRP synthesis to rescue STC in ageing. BDNF/TrkB signalling is known to be pro-LTP via its downstream enhancement of mTOR-dependent protein synthesis, along with CREB activation that drives expression of PRPs [150]. p75^NTR^ signalling is opposite in direction, diminishing synaptic strength in part through impairment of mTOR-dependent translation and CREB signalling, and its downregulation would enhance PRP synthesis [119,151]. HDAC3 inhibition promotes synaptic activity-dependent gene expression via nuclear factor kappa-light-chain-enhancer of activated B cells (NFκB) [152]. Shetty and Sajikumar [153] have discussed other possible factors leading to the loss of STC in ageing. Interestingly, these interventions can all enable STC expression in ageing even though they appear to affect different parts of the STC machinery. If tag setting processes are disrupted such that weaker stimulations do not produce effective tags for PRP capture, or that PRP synthesis and trafficking processes are impaired such that insufficient PRPs are available, we would expect these impaired processes to be limiting factors preventing STC expression under the current theory of STC. However, the findings here lead to a bold hypothesis: that tag setting and PRP capture processes can compensate for deficiencies in each other, such that strengthened PRP synthesis can be sufficiently captured by ageing-impaired synaptic tags for late-LTP expression in experimentally manipulated conditions. Cautious research is required before this hypothesis becomes more than theoretical conjecture, for it must be conclusively demonstrated whether certain processes influence only tag setting or PRP capture processes but not both. This can be difficult to argue for manipulations such as exogenous TrkB activation (since TrkB has been proposed to be a synaptic tag molecule [154]). Future experiments with more specific manipulations onto STC molecular machinery could aid to verify such a hypothesis.

### (e) Alzheimer’s disease

Alzheimer’s disease (AD), a neurodegenerative disease that severely impairs memory systems in patients, has been extensively studied under the neurological context [155,156]. Various hypotheses exist regarding the pathology of AD, and many of them have been associated with impairments in synaptic plasticity. The amyloid cascade is a well-known AD hypothesis that has been extensively studied from both pathological and therapeutic perspectives. According to the amyloid hypothesis, the aggregation of amyloid-β (Aβ) fragments—pathological metabolites of amyloid-precursor protein (APP)—results in neurotoxicity [157]. It has also been demonstrated that soluble Aβ, present at earlier stages of AD, can interfere with plasticity machinery, causing LTP impairments [158,159]. The exogenous and transgenic introduction of Aβ into mice models has been observed to inhibit LTP [160]. Tau aggregation has also been shown to impair memory functions and LTP [161]. Other mechanisms implicated in AD could also affect synaptic plasticity properties through calcium dysregulation that is not related to Aβ or tau [162,163].

Investigations into expression of associative plasticity in AD models, unsurprisingly, showed that STC is not expressed using the strong-before-weak protocol in APP/presenilin 1 (PS1) mice (APPswe/PS1dE9 double transgenic mice) or induced AD mice models (exogenous Aβ(1-42)) [164–168]. STC is also not expressed under the weak-before-strong protocol in APP/PS1 mice [169]. This is unsurprising considering that late-LTP itself is impaired in AD, and that the deficits in late-LTP would similarly prevent STC expression. Various interventions that target dysregulated pathways in AD that have downstream effects on plasticity machinery appear to be effective. For instance, applying glucose-derived carbon nanosphere-conjugated TTK21 ameliorates Aβ-induced LTP and STC deficits via restoration of AD-reduced CBP/p300 histone acetyltransferase activity [168]. Inhibition of G9a/GLP, a lysine methyltransferase complex, rescued LTP and STC in Aβ-induced AD and APP/PS1 mice models [165,166]. These methods target the AD-induced epigenetic state, characterized by increased histone deacetylation and methylation [170]. Plasticity changes in AD are known to be mediated by microRNAs (miRNA), and inhibition of a specific miRNA, miRNA-134-5p, rescued LTP and STC in Aβ-induced AD mice [167,171]. Another target in AD is Nogo-A, which is overexpressed in AD and involved in plasticity suppression. Inhibition of Nogo-A rescues LTP and STC in APP/PS1 mice [169]. Proteasome function is impaired in AD, and is instead involved in aberrant protein degradation that contributes to neurotoxicity [172,173]. Impairment of dysfunctional proteasome restores LTP and STC in an Aβ-induced AD model [174]. Metaplastic processes could be used towards ameliorating AD-related plasticity and STC impairments, and this has been demonstrated using RYR-priming to re-establish late-LTP and STC in APP/PS1 mice [164]. The lack of associativity in AD has also been demonstrated behaviourally, with loss of novelty-induced priming occurring before memory loss [175].

While there is a decent volume of work looking into the expression of STC in AD animal models, most of the research is focused on ameliorating pathways affected by AD. Nevertheless, some mechanistic interpretation can be done from biochemical and pharmacological work performed in those abovementioned papers. One particularly interesting result is the presence of late-LTD, but the absence of cross-tagging, in APP/PS1 mice [164]. For this to occur, the LTD tag and production of LTD-related PRPs must have successfully taken place, yet these LTD-related PRPs could not be captured at weakly tetanized synapses. Note that LTD induced via strong low-frequency stimulation is known to release PRPs that can be captured by LTP tags [48].

Hence, there are two possible explanations: either the tag setting process is impaired in LTP, or the PRPs synthesized by LTD are no longer compatible with LTP-set tags. Our current understanding of AD would give heavier weight to the first possibility, as that would also explain why LTP itself is impaired. However, many of the experimental interventions above appear to exert their effects through PRPs, with RYR through PKMζ; miRNA-134-5p inhibition through CREB and BDNF; G9a/GLP through enhanced protein synthesis and BDNF [164–167]. It is once again interesting how interventions that appear focused on one part of the STC machinery are sufficient in rescuing its expression even when both tagging and capture processes are required theoretically. This could support a similar hypothesis to ageing studies: tag setting and PRP capture processes can compensate for impairments in each other. Table 1 details evidence supporting this hypothesis that we have discussed thus far. Clear and cautious delineation of the exact STC processes impaired in AD should be performed before this line of reasoning can be taken any further.

**Table 1.** STC-relevant molecular machinery that is perturbed in or used to rescue STC impairments in pathologies. The involvement of each mechanism in tag setting (T) or PRP synthesis and capture processes (P) is specified. Question marks (?) denote uncertainty in the understanding of the specific influence that a mechanism has on STC.

pathological state	perturbed mechanism	STC process	ref	rescue mechanism	STC process	ref
juvenile stress	epigenetics: enhanced expression of histone methyltransferase G9a/GLP	P	[102]	epigenetics: G9a/GLP inhibition	P	[102]
sleep deprivation	decreased GluR1 expression	T	[114]	p75^NTR^ knockout	T, P	[118]
	decreased ERK activity	T	[114]	epigenetics: histone deacetylase inhibition	P	[122]
	impaired cAMP/PKA signalling	T	[113]			
	reduced mTOR-dependent protein synthesis	P	[115]			
	epigenetics: altered methylation state	P	[120]			
CRCI from hepatocellular carcinoma	unknown			antibiotics, to remove gut microbiota	?	[131]
ageing	altered NMDA receptor dynamics	T	[138]	zinc chelation	T	[145]
	altered calcium dynamics	T	[139]	p75^NTR^ knockout	T, P	[137]
	decreased dopamine receptor expression	P	[141]	epigenetics: HDAC3 inhibition	P	[146]
				BDNF/TrkB signalling	T^?^, P	[147]
AD	epigenetics: reduced histone acetyltransferase CBP/p300 activity	P	[168]	epigenetics: CBP/p300 activity enhancement	P	[168]
	miRNA dysregulation	?	[171]	miRNA-134-5p inhibition	?	[167]
	calcium dysregulation	T	[162,163]	Nogo-A inhibition	T, P	[169]
	Nogo-A receptor overexpression	T, P	[176]	RYR priming	T	[164]
	dysfunctional proteasome	T^?^, P	[173]	proteasome inhibition	T^?^, P	[174]

## 4. Conclusion

In the first half of this review, an overview of the extensive work that has been performed to understand STC molecularly, physiologically and behaviourally was laid out. In particular, the STC hypothesis provides explanatory power for memory phenomena such as flashbulb memories and prompts intriguing suggestions regarding the mechanism of information processing. In the second half, studies investigating STC within pathologies and how they led to an enhanced understanding of each pathology using the STC framework were discussed. Some of the benefits of analysing STC in pathologies were then elucidated. In particular, pathology-induced impairments to STC can be observed even when late-LTP can still be established. The increased sensitivity of STC to molecular disturbances is greatly beneficial in uncovering plasticity impairments at earlier stages of diseases. Interestingly, investigating STC in pathological states can in turn enhance our understanding of STC itself.

Our understanding of STC has grown significantly since its introduction 25 years ago. While there is still a need to elucidate the exact identities of the molecular players involved in this process, we now have sufficient knowledge to begin tapping on its incredible potential to refine our understanding of memory processes in everyday life and in disease. It is now the perfect moment to harness the explanatory power of STC in new contexts towards a more precise understanding of memory processes and pathologies.

## Data Availability

This article has no additional data.
